# The Gut Microbiome and Its Potential Role in the Development and Function of Newborn Calf Gastrointestinal Tract

**DOI:** 10.3389/fvets.2015.00036

**Published:** 2015-09-23

**Authors:** Nilusha Malmuthuge, Philip J. Griebel, Le Luo Guan

**Affiliations:** ^1^Department of Agricultural, Food and Nutritional Science, University of Alberta, Edmonton, AB, Canada; ^2^Vaccine and Infectious Disease Organization, University of Saskatchewan, Saskatoon, SK, Canada; ^3^School of Public Health, University of Saskatchewan, Saskatoon, SK, Canada

**Keywords:** gut microbiota, neonatal ruminants, gut development, mucosal immune system, enteric infections

## Abstract

A diverse microbial population colonizes the sterile mammalian gastrointestinal tract during and after the birth. There is increasing evidence that this complex microbiome plays a crucial role in the development of the mucosal immune system and influences newborn health. Microbial colonization is a complex process influenced by a two-way interaction between host and microbes and a variety of external factors, including maternal microbiota, birth process, diet, and antibiotics. Following this initial colonization, continuous exposure to host-specific microbes is not only essential for development and maturation of the mucosal immune system but also the nutrition and health of the animal. Thus, it is important to understand host–microbiome interactions within the context of individual animal species and specific management practices. Data is now being generated revealing significant associations between the early microbiome, development of the mucosal immune system, and the growth and health of newborn calves. The current review focuses on recent information and discusses the limitation of current data and the potential challenges to better characterizing key host-specific microbial interactions. We also discuss potential strategies that may be used to manipulate the early microbiome to improve production and health during the time when newborn calves are most susceptible to enteric disease.

## Introduction

**Figure 1:**
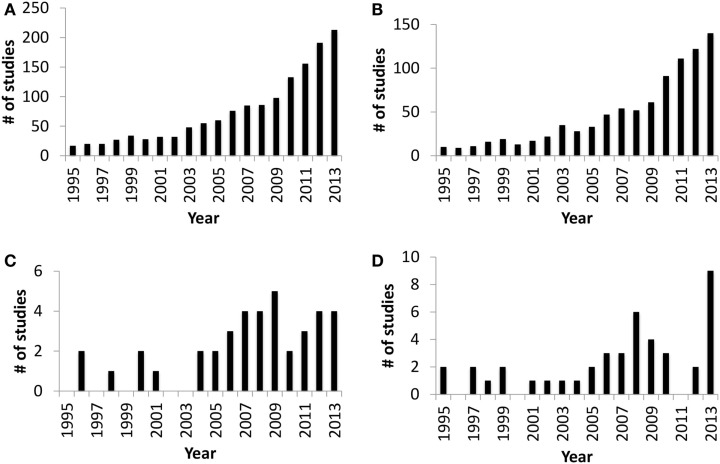
Number of publication entries in Medline (PubMed) trend* from 1995 to 2013. **(A)** Publication entries searched with query “gut colonization.” **(B)** Publication entries searched with query “gut colonization and human.” **(C)** Publication entries searched with query “gut colonization and ruminant.” **(D)** Publication entries searched with query “rumen colonization.” *Medline Trend, URL: http://dan.corlan.net/medline-trend.html.

The *in utero* sterile mammalian gastrointestinal tract (GIT) is rapidly colonized by an array of microbiota during and after birth. This process of colonization has been described as a co-evolution due to the two-way interaction between host and microbes (1). Host (luminal pH, food retention time in the gut, and immune defense mechanisms), microbial factors (adhesion, survival mechanisms under oxygen gradient, and mechanisms to obtain nutrients from the host), and external factors, such as maternal microbiota, delivery mode, diet, and antibiotic treatment during early life, all combine to influence gut colonization (2–4). The initial colonizers (*Streptococcus* and *Enterococcus*) utilize available oxygen in the gut and create the anaerobic environment required for strict anaerobic gut residents, such as *Bifidobacterium* and *Bacteroides* (2, 5, 6). *Bifidobacterium* and *Bacteroides* are two of the main gut bacteria present in the majority of human infants (3) that have a beneficial impact on mucosal immune system. The presence of *Bacteroides* in the gut plays a vital role in the development of immunological tolerance to commensal microbiota (7), while the composition of *Bifidobacterium* in the infant gut is linked to a reduced incidence of allergy (8). Therefore, neonatal gut colonization is a crucial period for the developing gut and naïve immune system (9, 10) and may have long-term health effects (5). Although research focused on understanding gut colonization of mammals has increased dramatically over the last decade (Figures 1A,B), there are still very few studies focused on domestic livestock species, especially ruminants (Figures 1C,D). Information is extremely limited on ruminant gut colonization, especially when focusing on the role of the microbiota in the early development of the GIT during the pre-ruminant period. Therefore, the present review builds on the information available for early colonization of the ruminant GIT to identify challenges in understanding the complex interaction between host and microbiome. We also use this information to speculate on possible strategies to engineer the microbiome and improve ruminant health and production.

## Gut Microbiota in Ruminants

Gut microbes of ruminants, mainly the rumen microbiota, provide 70% of their daily energy requirement via the fermentation of undigestible dietary substrates (11). Therefore, studies in ruminant gut microbiota have focused mainly on the rumen to understand how this microbiome impacts meat and milk production. Rumen microbiota consists of bacteria, archaea, protozoa, and fungi involved in the fermentation of complex carbohydrates, and their composition is influenced by a number of factors. For example, distinct microbial populations have been identified for the particle-attached, fluid-associated, and tissue-attached fractions of the rumen (12). Rumen microbial composition can also vary significantly depending on the ruminant species, diet, host age, season, and geographic region (13). Bacteria dominate the rumen microbiome and contribute mainly to the production of volatile fatty acids (VFAs) and microbial protein (14). Despite numerous human and mouse studies reporting the importance of early gut microbiota on host health, there are few attempts to understand the role of early gut/rumen colonization on GIT development or host health in neonatal ruminants. Furthermore, rumen/gut development and establishment of the microbiota have always been studied as separate aspects of ruminant biology and there have been few attempts to understand possible interactions between these two events.

### Rumen colonization in pre-ruminants

Colonization of pre-ruminant rumen was first studied using light microscopy and Gram-staining to visualize bacteria in the late 1940s (15, 16). In the 1980s, Gerard Fonty started to investigate the establishment of the rumen microbial community in lambs by using culture-dependent approaches and was the first to report age-dependent changes in the appearance of different microbial populations (17). Anaerobic bacteria dominate in the rumen of neonatal ruminants by the second day of life (10^9^ CFU/ml of rumen fluid) and the density of cellulolytic bacteria stabilized (10^7^ CFU/ml of rumen fluid) within the first week of life (17). This study revealed that the dominant bacterial species in the neonatal lamb rumen was different from those species colonizing the adult rumen. When the establishment of other microbial groups was investigated, their appearance was delayed until after bacteria were established (17). Anaerobic fungi and methanogens appear in the neonatal rumen between 8 and 10 days postpartum (17), while protozoa appear only after 15 days postpartum (18). Furthermore, comparison of conventionalized lambs with conventionally reared lambs suggested that the establishment of protozoa required a well-established bacterial population (18).

The early rumen microbiota consist of bacterial species from *Propionibacterium*, *Clostridium*, *Peptostreptococcus* and *Bifidobacterium* genera, while *Ruminococcus* species dominated the cellulolytic bacterial population (17). Restricted exposure of lambs to their dams or other animals also delayed the establishment of cellulolytic bacteria, when compared to lambs reared in close contact with their dams during the first few weeks of life (19). This observation revealed the important role of early environmental exposure for the establishment of a host-specific microbiota. Fonty and colleagues have also extended their studies to explore the establishment of tissue-attached (epimural) bacteria in the ovine rumen (20). Similar to the fluid-associated community, the complexity of the epimural community and homogeneity among individuals increased with increasing age (20). A recent study revealed, however, that the rumen epimural bacterial community in pre-weaned calves differs significantly from the content-associated community (21). This observation suggests that host–microbial interactions might play an important role in defining these two distinct microbial communities.

Rumen microbiota has a significant impact on pre-ruminant management, especially the weaning process, which depends on rumen development and the ability of the microbiome to ferment complex carbohydrates (22). The presence of VFAs (acetate, propionate, and butyrate) in the rumen plays an important role in rumen development, especially the development of rumen papillae (23). The fermentation of undigestible dietary substrates by rumen microbiota is the major source of VFAs in ruminants (11, 14), and it is generally believed that feeding a solid diet accelerates this process in pre-ruminants (22). Although the establishment of rumen microbiota has long been studied and their importance in the rumen development has been suggested, the mechanisms by which bacteria influence rumen development remain poorly defined. Moreover, culture-based studies can only identify around 10% of the total rumen microbiota, leaving the majority of the microbiome undefined (24).

Recently, enhanced molecular-based technologies, such as next generation sequencing (NGS), provide an excellent platform to identify both culturable and non-culturable microbes as well as characterizing their potential functions (25). It is now possible to generate a comprehensive profile of both microbial diversity and functions and explore potential associations between the microbiome and early rumen development. Using NGS, a comparison of the rumen bacteriome and metagenome in 2-week-old and 6-week-old calves, fed a milk replacer diet, revealed a taxonomically and functionally diverse rumen microbiome in pre-ruminant calves with significant age-dependent changes (26). This study revealed that *Bacteroidetes*, followed by *Firmicutes* and *Proteobacteria*, colonized in the rumen content of pre-weaned calves, which displayed age-dependent variations in their relative abundance. For example, the abundance of *Bacteroidetes* increased from 45.7% at 2 weeks to 74.8% at 6 weeks of age, despite calves receiving the same diet over time. Such age-related differences were more prominent at the bacterial genera level, where the predominant *Prevotella* (33.1%) at 2 weeks was replaced by *Bacteroides* (71.4%) at 6 weeks.

Since the study by Li and colleagues (26), there have been further studies analyzing changes in the composition of the rumen microbial community from birth to weaning. Rumen fluid or content was used as a proxy for the rumen microbiome and 16S rRNA amplicon-based sequencing approaches were used to identify and quantify bacteria (21, 27–29). These studies revealed marked heterogeneity in the rumen bacterial composition of individual animals immediately postpartum, but greater similarity in bacterial composition was observed with increasing age (26–29). There were, however, a number of discrepancies in terms of rumen bacterial composition when comparing among studies. For example, Jami and colleagues (27) reported a higher abundance of *Streptococcus* belonging to the phylum *Firmicutes* in 1–3-day-old calves. In contrast, Rey and colleagues (28) reported a higher abundance of *Proteobacteria* in 2-day-old calves. Furthermore, both Jami et al. (27) and Rey et al. (28) reported a higher abundance of *Bacteroides* in rumen fluid at 2 weeks of life, while Li and colleagues (26) observed a greater abundance of *Prevotella* in rumen content. Targeting different variable regions of 16S rRNA gene (V1/V2 versus V3/V4) for amplicon-based sequencing and differences in the environment, in which these calves were raised, may have influenced the apparent bacterial composition of rumen fluid.

A study comparing content-associated versus epimural bacterial populations in 3-week-old calves revealed that bacterial phylotypes belonging to *Bacteroidetes* (43.8%) and β-*Proteobacteria* (25.1%) dominated the epimural community. In contrast, phylotypes from *Bacteroidetes* (54.8%) and *Firmicutes* (29.6%) dominated the rumen content-associated community (21). Using 16S rRNA amplicon-based sequencing, temporal changes in the epimural bacterial community have also been reported in goat kids during the first 10 weeks of life (30). The predominant *Proteobacteria* (>85%) during the first week of life were gradually replaced by an increasing abundance of *Bacteroidetes* (~10%) and *Firmicutes* (>15%) (30). Similar to previous culture-based approaches, these recent studies have confirmed that dynamic changes occur in the rumen bacterial community during early life, with significant differences between the epimural and fluid-associated communities in the pre-weaned rumen.

**Figure 2:**
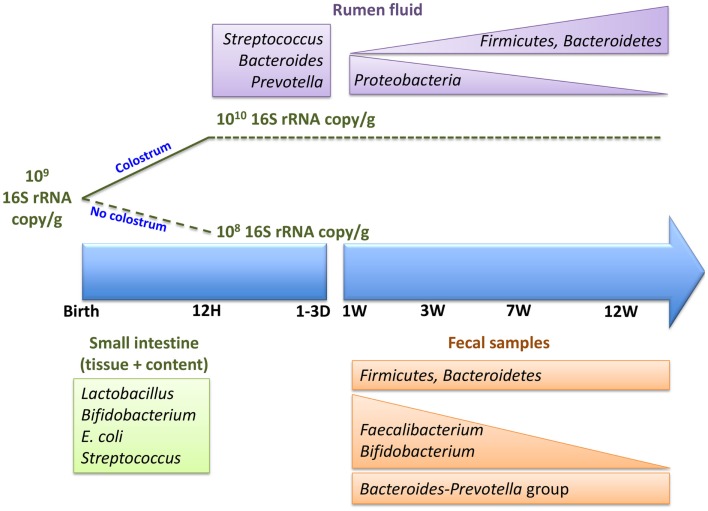
**Colonization of neonatal calf rumen/gut, immediately postpartum and within the first 12 weeks of life**.

Associated with the age-dependent changes in rumen microbial composition (Figure 2), there are also changes in the activity of the rumen microbiota. These functional changes occur in the absence of dietary changes during the first 6 weeks of life (26). Currently, this is the only study using a metagenomic approach to assess the metabolic potential of pre-ruminant rumen microbiome. Li and colleagues (26) revealed that ATP-binding cassette family transporters are more abundant at 2 weeks than 6 weeks of age but TonB-dependent receptors are more abundant at 6 weeks. Glycoside hydrolases (GH2, GH3, GH42, and GH92), which breakdown complex carbohydrates, were also detected in the pre-ruminant rumen, even when the diet did not contain complex carbohydrates. These observations suggest that early rumen microbiota has the capacity to ferment dietary fiber prior to being exposed to this material. Moreover, a recent study investigating the activity of the early rumen microbiome revealed that VFA production and xylanase and amylase, enzymes that breakdown complex carbohydrates, were active within 2 days postpartum (31). The observed glycoside hydrolase activity, in conjunction with VFA production, reveals establishment of a metabolically active adult-like microbiome in the neonatal rumen prior to exposure to appropriate dietary substrates. Thus, the establishment of metabolically active microbiome may occur along with the transfer of microbiome from the dam to newborn calf and the colonization of a species-specific microbiome.

Diet is one of the main factors that influences the composition of gut microbiota and may also play an important role in the observed temporal changes of the rumen microbiome in neonatal calves (27, 28). The rumen content of 3-week-old calves fed milk replacer, supplemented with a calf starter ration (20% crude protein, 3% crude fat, and 5.7% crude fiber), contained a similar abundance of *Prevotella* (15.1%) and *Bacteroides* (15.8%) (21). Calves that received milk replacer only, however, displayed a shift in the predominant rumen content-associated bacteria from *Prevotella* to *Bacteroides* (26) within the first 6 weeks of life. Thus, the observed similar abundance of these two bacterial genera in 3-week-old calves fed milk supplemented with calf starter suggests that the age-dependent shift in the dominant bacteria may have been triggered by the dietary supplement that contained fiber. In general, it is believed that the introduction of solid diet plays a key role in promoting the establishment of rumen microbiota as milk bypasses the rumen to enter the abomasum (22). Moreover, pre-weaning diet and feeding methods have been reported to have more pronounced and long-lasting impacts on rumen microbial composition (29, 32, 33). Altering feeding practices during the pre-weaning period were reported to significantly alter methanogen composition after weaning (32) as well as the density of bacteria and protozoa in pre-weaned lambs (33). Therefore, managing pre-weaning feeding may be as important as managing feeding during the weaning period in terms of microbiota establishment as well as development of the microbial fermentation capacity of the rumen.

Currently, characterization of the rumen microbiota is based primarily on the sequencing of DNA, which represents both active and dead microbiota. Therefore, the use of RNA-based metatranscriptome approaches may provide a better understanding of the biological activity of the early rumen microbiome. Understanding the activity of the rumen microbiota may help designing multidisciplinary approaches to engineer the early rumen microbiome with the objective of promoting both rumen development and function that better supports the critical transition that occurs when ruminants are weaned.

### Intestinal tract colonization in pre-ruminants

Early studies on bacterial colonization of the pre-ruminant intestine focused primarily on pathogenic *Escherichia coli* in calves and described the pathogenesis of neonatal diarrhea (34–37). Microscopic imaging revealed that pathogenic *E. coli* preferably attached to and effaced the mucosal epithelium in the ileum and large intestine, but not the duodenum and jejunum of neonatal calves (36). Feeding of probiotic strains isolated from the intestine of calves reduced enteric colonization of pathogenic *E. coli* O157:H7 in pre-weaned calves (38). Furthermore, the administration of *Bifidobacterium* and *Lactobacillus* to newborn calves during the first week of life increased weight gain and the feed conversion ratio, while decreasing diarrhea incidences (39). These effects were most pronounced in pre-weaned calves than weaned calves (39), suggesting the probiotic supplements are more effective when the gut microbiota is being established and less effective when the microbiome has stabilized.

Supplementation of *Lactobacillus* in young calves was also reported to increase the total serum immunoglobulin G concentration (40), providing evidence of a host–microbiome interaction that may influence calf health. More recently, supplementation of newborn calves with prebiotics (galactooligo saccharides) was associated with an increased abundance of *Lactobacillus* and *Bifidobacterium* in the colon of 2-week-old calves (41). However, this effect was less pronounced in 4-week-old calves (41), suggesting that as with probiotics, it may be easier to manipulate the microbiome during the early colonization period (39). In an attempt to reduce antibiotic usage during the pre-weaning period, studies continue to investigate the impact of both probiotics and prebiotics on calf growth and health (42). The full impact of these approaches on gut microbial colonization and composition throughout the pre-ruminant period has yet to be understood and studies are lacking on how altering the gut microbiome may impact mucosal immune defenses in the GIT.

In 1965, Williams Smith used culture-dependent approaches for the first time to study bacterial colonization in the pre-ruminant GIT, beginning immediately postpartum. He reported colonization by *E. coli* and *Streptococcus* in all gut regions (stomach, small intestine, and cecum) of calves within 8 h after birth, while *Lactobacillus* colonization was only observed 1 day after birth. *Lactobacillus* then predominated throughout all regions of the GIT tested within the first week (43). *Bacteroides* were observed only in the cecum and feces after the second day of life (43). The colonization of *Clostridium perfringens*, previously known as *Clostridium welchii*, was also observed in the cecum within 8 h after birth; however, it was not detected in other gut regions until 18 h after birth (43). This study suggested that the newborn GIT was first colonized by facultative anaerobes, which then created the anaerobic conditions required for colonization by obligate anaerobic gut microbiota, such as *Lactobacillus* and *Bacteroides*. A similar evolution of bacterial colonization of the GIT has been reported for other newborn mammals (6).

Subsequent studies have revealed a higher abundance of *Bifidobacterium* and *Lactobacillus* in fecal samples and throughout the GIT of newborn calves (44, 45). A higher abundance of *Bifidobacterium* in 3–7 days old calves was also associated with a lower abundance of *E. coli* (44). More recently, culture-independent approaches have been employed to better understand the diversity and abundance of bacteria throughout the neonatal ruminant GIT (46, 47). RNA-based, sequence-specific rRNA cleavage analysis of bacteria throughout the first 12 weeks postpartum revealed a higher abundance of the *Bacteroides*–*Prevotella* and *Clostridium coccoides*–*Eubacterium rectale* groups in the feces of dairy calves (46). *Faecalibacterium* was one of the most abundant bacteria in 1-week-old calves (21.7%), but then declined with increasing calf age (46). *Ruminococcus flavefaciens* and *Fibrobacter*, fibrolytic bacteria, were only observed after 5 weeks postpartum, while *Streptococcus* and *Lactococcus* could not be detected after the fifth week (46). These studies confirmed that there were significant age-dependent changes in the composition of the GIT microbiome and revealed substantial differences between the rumen and lower GIT microbiome.

Regional variations in bacterial phylotypes richness, diversity, density, and composition throughout the GIT of newborn calves have been described, using both culture-dependent and independent approaches (21, 45, 48, 49). When bacterial populations throughout the GIT of 20-week-old calves were analyzed, *Bifidobacterium* and *Lactobacillus* displayed greater survival of stomach passage than coliforms and *E. coli* (45). The density of these beneficial bacteria was high throughout all GIT regions (rumen, abomasum, duodenum, jejunum, cecum, and colon) of the 20-week-old calves (45). Using culture-independent approaches, higher bacterial phylotype richness was observed in the rumen and large intestinal regions than the small intestinal regions of lambs and calves (21, 48, 49). Collado and Sanz (48) reported, however, a similar bacterial richness throughout the GIT, when using a culture-dependent approach. This observation is consistent with there being many more unculturable bacterial species in the rumen and large intestine than the small intestine. A longer retention time, higher availability of nutrients, and reduced scrutiny by the host mucosal immune system have all been suggested to contribute to the increase in bacterial diversity and density in the rumen and large intestine of mammals (1).

When bacterial composition throughout the GIT is explored, the rumen and large intestinal regions consist primarily of *Bacteroidetes* and *Firmicutes*, while >95% of the bacteria in the small intestine contents are composed of *Firmicutes* (21). In contrast, the mucosa-associated bacterial community in the small intestine is composed of primarily *Bacteroidetes*, *Firmicutes*, and *Proteobacteria*, including 17 genera that are unique to this region of the GIT (21). The presence of bacteria unique to the small intestine (21) suggests that fecal sample-based studies do not reveal the true GIT microbiome and may not reveal important regional host–microbial interactions. A recent study in human infants reported similar observations and it was also concluded that feces was not representative of host–microbiota interactions throughout the gut (50).

There is increasing evidence that mucosa-attached microbiota are significantly different from those associated with ingesta and present in the intestinal lumen. Collado and Sanz (48) first studied mucosa-attached bacteria and reported that *Bifidobacterium* and *Lactobacillus* were predominant throughout the GIT (rumen, duodenum, and colon) of calves (9–11 months) and lambs (6–9 months). They did not, however, compare mucosa-associated versus intestinal content communities. Studies by Malmuthuge and colleagues (21, 49) compared mucosa-attached and content-associated bacterial communities throughout the GIT of calves and reported that at 3 weeks of life, distinct mucosa-attached bacterial phylotypes had been established. Furthermore, bacterial richness in mucosa-attached communities, especially in the ileum, was higher than the content-associated community (49). These distinct and richer mucosa-attached bacterial communities were subsequently confirmed by using pyrosequencing of 16S rRNA gene amplicons (21). Although the majority of mucosa-attached bacteria could not be assigned at a genus level, the use of a NGS approach provided a greater understanding of region- (rumen, small intestine, and large intestine) and sample type- (content and mucosa) specific bacteria throughout the GIT of pre-weaned calves (21).

Based on the previously cited studies, it is clear that the composition, diversity, and richness of rumen and intestinal microbiota in pre-weaned ruminants can vary depending on various factors, such as age, diet, feeding method, feed additives, sampling location (content, mucosa, and feces), and gut region (rumen, large intestine, and small intestine) (Table 1; Figure 2). Furthermore, variation in microbial composition among individual animals is higher in young than adult ruminants (27). The high variation in bacterial diversity and density (27, 49) among individual ruminants during early life also suggests that the gut microbiome may be more easily changed at this time of life than in adults. This may explain why probiotics and prebiotics have been reported to have a much greater effect in young animals than older calves (39, 41). Of particular interest are the recent studies conducted by Abecia and colleagues, which revealed long-lasting consequences when dietary interventions were used to manipulate the rumen microbiota in young calves. Thus, a much greater understanding of early gut microbial colonization and the factors influencing establishment of microbiota may provide the basis for rational strategies to manipulate the gut microbiome and improve the growth and health of ruminants throughout the entire production cycle.

**Table 1:** **Factors influencing pre-weaned calf rumen/gut microbiota**.

Factor	Study
Age	(17, 26–28, 30, 46, 47, 49)
Diet (colostrum, calf starter)	(28, 29, 32, 33, 46, 51, 52)
Feeding method (suckling, bottle feeding)	(53)
Probiotic, prebiotics	(39, 41)
Exposure to dam	(19, 53)
Sample site	(21, 43, 48, 49)
Sample type (fluid, content, mucosa)	(20, 21, 49)
Host (individuality)	(27)
Infections	(47)

## Influence of Microbiome on Gut Development and Mucosal Immune Functions

Gut microbiota are essential for the development and differentiation of the intestinal mucosal epithelium as well as the mucosal immune system (54). Most of our knowledge regarding host–microbiome interactions in the GIT has been obtained from a variety of mouse models. Comparisons between gnotobiotic and conventionally reared mice revealed decreased development of the intestinal epithelium and the mucosal immune system in the absence of gut microbiota. Thickness of the mucus barrier is reduced in germfree mice, but administration of microbe-derived lipopolysaccharides and peptidoglycans to the colonic mucosal surface stimulated mucus production and within 40 min restored the thickness of the mucus layer to that of conventional mice (55). This observation supports the conclusion that the gut microbiota is essential for the secretion of intestinal mucus, an important physical barrier throughout the GIT. In addition, the generation rate of epithelial cells in germfree mice is lower than that of the conventionally raised mice (56), revealing the importance of gut microbiota for maintaining intestinal epithelial cells proliferation and ensuring recovery of the mucosal barrier following injuries.

The presence of gut microbiota in mice is also necessary for the development of secondary lymphoid structure, such as Peyer’s patches (PPs), mesenteric lymph nodes, and isolated lymphoid follicles (54). The establishment of host-specific microbiota, especially bacterial species belong to phylum *Firmicutes*, is essential for the development of a variety of intestinal immune cells (57). For example, when human microbiota colonized the mouse intestine there were low numbers of CD4^+^ and CD8^+^ T cells, and fewer proliferating T cells and dendritic cells when compared to mice colonized with mouse microbiota (57). Interestingly, the immune cell profile of human microbiota colonized mice was similar to that of germfree mice (57), suggesting the presence of a host-specific microbiota is fundamental for mucosal immune system development. Thus, host–microbial interactions in the developing gut of newborn animals must be studied within relevant host species to accurately understand the role of early microbiota on gut development.

In ruminants, development of mucosa-associated lymphoid tissues (MALTs) in the GIT begins *in utero* and there is active proliferation of B cells in lymphoid follicles of the PP in the complete absence of the gut microbiome (58, 59). Furthermore, oral delivery of antigens *in utero* has confirmed that these MALTs are fully functional and can generate specific immune responses with the production of secretory IgA (60). In the absence of an *in utero* infection, however, the appearance of IgG^+^ and IgA^+^ cells in PPs is delayed until after birth (59). Since immunoglobulin class switching occurs in the germinal centers of PPs (54), this suggests that the full development of germinal centers requires exposure to the gut microbiota. However, information regarding the role of the gut microbiota in the early postnatal development of MALT in ruminants is scarce. There is a single report that preventing exposure of the ileal PPs to gut microbiome results in premature involution of lymphoid follicles in the PPs of newborn lambs (61). However, restoration of the gut microbiome at 4 weeks after birth reversed lymphoid follicle involution in the ileal PPs (61). Thus, the gut microbiome appears to provide critical signals that maintain the production of the pre-immune B cell repertoire. It remains to be determined whether specific microbial species may influence the selection of this immunoglobulin repertoire or if this interaction is restricted to an interaction with innate immune receptors.

The host uses pattern recognition receptors, such as toll-like receptors (TLRs), to recognize the commensal microbiota and maintain intestinal homeostasis (62). Activation of TLR signaling by intestinal tissue invading pathogens generally stimulates inflammatory responses. In contrast, commensal microbiota activation of TLR signaling promotes the production of interleukin 6 and tumor necrosis factor that protect intestinal epithelial cells against injury (62). Therefore, commensal microbial recognition by mucosal TLRs is crucial for the maintenance of intestinal homeostasis and protection of the gut from injuries. The expression of TLRs in the blood of infants (63) was downregulated with increasing age, while memory T cells, such as CD4^+^ and CD8^+^, increased in number (63). These changes are consistent with a decrease in innate immune responses that is balanced by an increase in adaptive immune responses with increasing age. Downregulation of innate immune responses with increasing age has been suggested as one mechanism by which the host avoids unnecessary inflammatory responses to commensal microbiota (63). Similar results have been reported when analyzing the intestinal immune system of calves (64, 65). The expression of mucosal TLR genes was downregulated in weaned calves when compared to pre-weaned calves (65). In contrast, total leukocytes including, CD3^+^, CD4^+^, and CD8^+^ T cells, increased in the jejunal and ileal mucosa of calves with increasing age (64). Moreover, a negative correlation was observed between the expression of mucosal TLRs and mucosa-attached bacteria, suggesting a possible link between the gut microbiota and the observed age-related changes in the mucosal immune responses (65). However, the mechanism by which gut microbiome colonization affects this shift of mucosal and systemic immune responses from innate to adaptive remains to be defined. There is, however, emerging evidence that microbial colonization is associated with substantial changes in the transcriptome of the bovine intestine during the first week of life (66). Transcriptome changes occurred at the level of miRNA and significant correlations were identified between the gut microbiome and these transcriptome changes.

Experiments with the mouse model have clearly demonstrated the importance of gut microbiota in the development of both innate and adaptive components of the mucosal immune system as well as development and maintenance of the intestinal epithelial barrier. Increased susceptibility to enteric infections in gnotobiotic and antibiotic treated mice may also be due to the underdeveloped mucosal immune system and epithelial barrier (54). The immunologically naïve neonatal GIT and the colonizing microbiota undergo a rapid co-evolution during early life and these interactions may be crucial in determining the susceptibility of the neonate to enteric infections. Pre-weaned ruminants are highly susceptible to a variety of viral and bacterial enteric infection within the first few weeks of life (67). Therefore, a thorough understanding of early gut microbiota and its role in regulating and directing early development of the mucosal immune system is essential to improving the health of young calves and reducing susceptibility to enteric infections.

## The Commensal Microbiome and Enteric Infections in Young Ruminants

Neonatal diarrhea is the major cause of death in pre-weaned calves and accounts for >50% of calf deaths in the dairy industry (67). Establishment of the gut microbiome within the first 7 weeks of life and an association with calf health and growth (neonatal diarrhea, pneumonia, and weight gain) was recently reported (47). Bacterial diversity was lower in calves with pneumonia and neonatal diarrhea when compared to healthy calves (47), suggesting a possible link between gut microbiota and host health. The authors speculate that antibiotic treatment may have been one factor influencing the gut microbiome in pneumonic calves. Furthermore, colonization by enteric pathogens may be responsible for the observed dysbiosis in gut microbiota during neonatal diarrhea (47). Increased fecal bacteria diversity was also associated with increased weight gain in healthy calves, while a high abundance of *Faecalibacterium* during the first week of life was associated with a lower incidence of diarrhea in calves after the fourth week of life (47). Thus, it is difficult to determine if changes in the fecal microbiome were a consequence of prior disease and associated therapeutic interventions or if colonization of the GIT by specific commensal bacteria had a beneficial effect in terms of disease resistance.

Uyeno and colleagues (46) also reported a high abundance of *Faecalibacterium* in the feces of 1-week-old calves and their abundance was higher in the large intestine compared to the small intestine of 3-week-old calves (21). *Faecalibacterium prausnitzii*, one of the main butyrate producers in the large intestine, displayed a negative association with calf diarrhea incidences (47), suggesting the high prevalence of this species during early life may decrease susceptibility to enteric infections. *F. prausnitzii* also plays a pivotal role in maintaining intestinal homeostasis by promoting anti-inflammatory responses and has been shown to decrease in prevalence in patients with inflammatory bowel disease (68). Inflammatory bowel disease was also associated with a reduced prevalence of *Bifidobacterium* (68), suggesting that these two bacterial groups may have important roles in maintaining intestinal homeostasis and preventing enteric infections. Thus, it will be important to further explore the potential role of such beneficial bacteria in the early gut development and their capacity to promote host health.

Poor management of colostrum feeding in newborn calves is one of the main triggers of neonatal calf diarrhea. Feeding calves with highly contaminated (bacteria > 106 CFU/ml, coliform > 103 CFU/ml) and of low quality (IgG < 50 mg/ml) colostrum (69), poor surveillance of calves born at night, and relying on dams to feed colostrum (70) are some of the major risk factors currently contributing to poor neonatal calf health in the North American dairy industry. Although the importance of timed feeding of high quality colostrum for passive transfer of immunity has been well studied (71), the influence of colostrum on gut microbial establishment and susceptibility to enteric infection in young ruminants is not clearly understood. A recent study revealed that feeding colostrum within 1 h postpartum facilitated bacterial colonization of the small intestine within the first 12 h postpartum. Calves-fed colostrum achieved bacterial numbers similar to older calves [10^10^ 16S rRNA gene copy/g of intestinal sample (49)], but significantly fewer bacteria were observed in the intestine of calves deprived of colostrum (52). Furthermore, when comparing to colostrum-deprived calves at 12 h postpartum, there was a significant increase in the prevalence of *Bifidobacterium* and a decreased prevalence of *E. coli* in the mucosa-attached communities of calves fed either heat-treated or fresh colostrum (52). Changes in the abundance of mucosa-attached *Bifidobacterium* and *E. coli* populations were most pronounced when calves were fed heat-treated colostrum versus fresh colostrum (52). Heat treatment (60°C, 60 min) decreases the density of total bacteria including pathogens present in colostrum, which has been suggested to decrease neonatal diarrhea in calves (71). The results from Malmuthuge and colleagues (52), however, suggest that timed feeding of high quality colostrum has a direct effect on bacterial colonization of the bovine small intestine, in particular the mucosa-attached community that is in close contact with the host mucosal immune system. Establishing a bacterial population dominated by beneficial bacteria may suppress colonization of enteropathogens (72) immediately postpartum and provide protection against enteric infections in young ruminants with a naïve immune system. Further investigations are necessary to also understand how a *Bifidobacterium*-dominated early gut microbiome may influence host performances (weight gain, resistance to enteric infections) within the first few weeks and identify the mechanisms by which the commensal microbiome alter both enteric health and general physiology.

## Manipulation of the Early Gut Microbiome to Improve Health and Production

Manipulation of gut microbiota by feeding microbes, probiotics, or prebiotics has been widely studied in livestock animals as a strategy to improve production and health through altering rumen fermentation and preventing pathogen colonization (24, 42). Direct-fed microbials have been shown to decrease rumen acidosis in cattle, increase milk production in cows, and decrease fecal shedding of *E. coli* in calves (73). These direct-fed microbials may prevent enteropathogen colonization of the gut by either competing for nutrients, space in the gut environment, or producing antimicrobial substances (73). *Megasphaera elsdenii* modifies ruminal fermentation and decreases ruminal acidosis by utilizing lactic acid produced in the rumen (73). However, most of these outcomes are limited to a relatively short interval following feeding (24) or are effective only in pre-weaned calves (39), suggesting that these manipulations are either temporary or need to be instituted within a defined developmental period. Moreover, it is essential to know how the autochthonous gut microbial population responds to these dietary manipulations and how their compositional changes influence overall gut metabolic and immune functions. It may also be important to determine if developing probiotics or direct-fed microbials, based on *Faecalibacterium* and *Bifidobacterium* that have already been linked to calf health, provides a more effective or long-lasting effect. The establishment of host-specific bacteria is crucial for the development of mucosal immune system, especially for the differentiation and proliferation of T cell populations (57). Thus, there would be substantial value in both isolating and testing bacteria within the same host species that might provide the basis for the developing microbial manipulation techniques.

## Conclusion

Interactions between host and gut microbiota have been explored extensively in humans and mice but these investigations are still in their infancy in ruminants (Figure 1). However, the studies reviewed to date are generating promising results, describing GIT microbial composition (Figure 2) and functions in greater depth and identifying factors that significantly influence microbial establishment. It is also notable that recent results are based primarily on nucleic acid sequencing, which may be limited by sampling location, the type of sample collected, extraction methods, sequencing depth, and the analysis pipeline used. In addition, the taxonomic and functional identification of the rumen/gut microbiome is dependent on existing databases and identified organisms and functions are remaining unclassified at lower taxonomic levels and at the level of protein coding genes. Single cell genome sequencing and more comprehensive databases for the ruminant gut microbiome are vital to understanding their role in host development.

A substantial step forward in being able to explain the role of the gut microbiome in host physiology would be to understand the metabolic capacity of the early microbiome. Metabolic functions of the rumen microbiota appear to be highly redundant, which may be essential to ensure optimum fermentation of ingested substrates. Therefore, isolation of metabolically active rumen microbiota may be important to further our understanding of their roles in monocultures and mixed populations. This information will provide the basis for future strategies designed to manipulate the microbiome and improve both production and health.

Finally, there is a substantial need to develop ruminant animal models that can be used to investigate the effects of controlled changes in the gut microbiome on both host mucosal immunity and host metabolism. The rearing of gnotobiotic calves is limited by large technical and financial barriers and to date studies have been limited to changes in diet or the feeding of pre- or probiotics and subsequent sampling of rumen or fecal microflora. The challenge is to develop animal models that allow us to ask questions regarding microbiome changes within specific regions of the GIT and to analyze local effects on mucosal immune and barrier function. The use of a surgically isolated intestinal segment model in fetal lambs (61) provided an elegant model system to create a localized gnotobiotic environment in the GIT of a developmentally normal animal. A similar model system was developed in newborn calves to study the effects of a persistent enteric bacterial infection (74). Thus, it should now be possible to manipulate local exposure to the microbiome and analyze the effects on neonatal mucosal immune system and barrier development. A critical question to be addressed is whether dysbiosis of the microbiome during colonization of the newborn GIT has long-term effects, both locally in the GIT and systemically, that impacts the health and production of animals. If long-term effects are observed, then it will be important to determine if restoration of the complex microbiome, or specific bacterial species, can effectively reverse the effects of early microbial dysbiosis.

## Conflict of Interest Statement

The authors declare that the research was conducted in the absence of any commercial or financial relationships that could be construed as a potential conflict of interest.
